# The association between digital peer support and mental health outcomes in university students: a cross-sectional study

**DOI:** 10.3389/fpubh.2026.1595794

**Published:** 2026-06-17

**Authors:** Iftikhar Ahmed Charan, Shazia Soomro, Syed Esam Mahmood, Ayed A. Shati, Youssef A. Alqahtani, Marium Aftab

**Affiliations:** 1 School of Public Administration, Shandong Technology and Business University, Yantai, Shandong, China; 2 Department of Sociology, School of Sociology and Political Science, Anhui University, Hefei, Anhui, China; 3 Department of Family and Community Medicine, College of Medicine, King Khalid University, Abha, Saudi Arabia; 4 Department of Child Health, College of Medicine, King Khalid University, Abha, Saudi Arabia; 5 Boston Neurobehavioral Associates, New Bedford, MA, United States

**Keywords:** anxiety and depression, cross-sectional study, digital peer support, socioeconomic factors, university students

## Abstract

**Background:**

The growing prevalence of mental health issues among university students underscores the need to explore factors associated with their wellbeing. This study investigates the influence of socio-demographic factors and perceived digital peer support on self-rated general physical health and mental health outcomes of undergraduate students. The research aims to examine how these factors are associated with student health and explore the potential role of perceived digital peer support in mental health.

**Methods:**

A cross-sectional study was conducted with 230 university students. The Generalized Anxiety Disorder-7 (GAD-7) scale was used to assess anxiety symptoms, and the Patient Health Questionnaire-9 (PHQ-9) was used for depressive symptoms. Data on perceived digital peer support, self-rated general physical health, and socio-demographic characteristics were also collected. Chi-square tests with effect sizes (Cramer's V) and multivariate binary logistic regression were used to adjust for confounders.

**Results:**

High rates of mental health symptoms were observed, with 43.5% of participants reporting moderate-to-severe anxiety and 53.5% moderate-to-severe depression. Perceived digital peer support was significantly associated with a lower rate of both anxiety (χ^2^ = 4.760, *p* = 0.029) and depression (χ^2^ = 4.708, *p* = 0.026) in bivariate analyses. Multivariate models confirmed digital peer support was an independent protective factor against anxiety (AOR = 0.55, 95% CI = 0.31, 0.97) and depression (AOR = 0.52, 95% CI = 0.29, 0.93). Lower personal income is consistently associated with higher odds of both anxiety (AOR = 2.10, 95% CI = 1.05, 4.21) and depression (AOR = 2.25, 95% CI = 1.12, 4.52).

**Conclusion:**

Perceived digital peer support is significantly associated with lower odds of moderate-to-severe anxiety and depression among university students, particularly for those with lower incomes. These findings suggest an association that warrants further exploration for campus mental health initiatives, especially for economically disadvantaged students.

## 1 Introduction

The global burden of mental health challenges, particularly among young adults, underscores the need to identify accessible sources of support. Peer support the mutual exchange of assistance based on shared experiences, respect, and understanding, is recognized as a mental health recovery and promotion (1). The evidence suggests that integrating peer support with traditional services can improve engagement and outcomes (2). With the proliferation of digital technology, the delivery of peer support has expanded into online spaces (3), creating new avenues for connection labeled as digital peer support (4).

University students represent a population at heightened risk for anxiety and depression, and perceived declines in physical health due to academic, social, and financial pressures (5). Digital peer support, facilitated through social media, messaging apps, or dedicated platforms, may serve as a contemporary and accessible resource for this demographic (6, 7). Preliminary research indicates that engagement with digital peer networks is associated with aspects of psychological wellbeing (8). However, the specific association between perceived digital peer support and common mental health outcomes like anxiety and depression in a general student population remains underexamined (9).

Furthermore, the potential link between digital peer support and self-rated physical health is poorly understood (10). Mental health and physical health are deeply interconnected (11, 12). Most of the research on digital peer support focuses on clinical populations or specific mental illnesses (13). This leaves a significant gap in understanding its role for otherwise healthy individuals, particularly young adults navigating normative stressors (14). It is also crucial to account for socio-demographic factors, such as personal income, which are strongly associated with health disparities and may influence both the perception of support and health outcomes (15).

This cross-sectional study aims to investigate the association between perceived digital peer support and symptoms of anxiety and depression, as well as self-rated general physical health, among university students, while adjusting for key socio-demographic characteristics. By focusing on a non-clinical student sample, this research seeks to address a gap in the literature concerning the role of digital peer support in general students' wellbeing.

## 2 Materials and methods

### 2.1 Study design, participants, and settings

This study employed a cross-sectional, observational design to investigate the associations between perceived digital peer support and mental health outcomes among a population of undergraduate university students. The primary aim was to assess whether varying levels of self-reported digital peer support were correlated with symptoms of depression and anxiety, and psychological distress. We explicitly accept that the cross-sectional nature of this data allows correlations to be identified but cannot provide evidence for the causation of peer support in mental health. We used a convenience sampling approach to participants from the entire undergraduate student body in spring 2024. All potential participants were emailed the invitation through official university contacts. An email included a direct link to the study fact sheet and digital questionnaire. The survey was administered for the purposes of data collection over five weeks, closing at the end of the semester.

The survey link was clicked, and the questionnaire was initiated by about 350 from a potential target population of 5,000 or so eligible undergraduate students. Two hundred and thirty completed valid questionnaires were received, a participation rate of 4.6% from the general population, with a completion rate of 65.7% among those who initiated the survey. This study was ethically approved by the Institutional Review Board (IRB) at King Khalid University. The study adhered to the principles outlined in the Helsinki Declaration, and informed consent was obtained from all participants before inclusion. A digital consent form, which included information about the study's objectives, procedures, potential risks, benefits, and voluntary nature of participation, was presented to participants at the beginning of the survey.

Inclusion was limited to individuals who were currently enrolled as undergraduate students at the university, as verified by a screening question at the survey start. The primary exclusion criterion was the provision of an incomplete survey response. We defined an incomplete response as one missing more than 20% of the items on any of the primary psychological scales (PHQ-9, GAD-7, K6). A total of 70 respondents were excluded from the initial 300 for this reason. For the remaining 230 participants, minor missing data (less than 20% of items on a scale) was handled by calculating a prorated score for that scale based on the mean of the answered items, provided the participant had completed at least 80% of the scale items (16, 17).

### 2.2 Sample size

To ascertain the minimal sample size needed, an *a priori* power analysis was carried out using G^*^Power software (version 3.1). The analysis indicated that a sample size of 160 participants was needed for a multiple linear regression analysis with an expected medium effect size (f^2^ = 0.15), a significance level of (α) 0.05, statistical power of 95%, and eight predictors (including the primary independent variables and important demographic covariates). Our final sample of 230 undergraduate students (54.3% female; SD = 3.67), therefore, exceeds this minimum requirement, providing adequate power to detect significant effects. The participants represented diverse socioeconomic and educational backgrounds, allowing the results to be generalized more confidently across this population.

### 2.3 Measurements

#### 2.3.1 Perceived digital peer support

“Perceived Digital peer support” was defined as the subjective appraisal of available emotional, instrumental, and informational support from other students via digital platforms (e.g., messaging apps, social media, online university forums). This construct was measured using an adapted version of the five-item “Peer Personal Support” subscale from the Classroom Life Scale (18). To situate the items for online peer interactions, we iteratively reworded each item. The modification was that references to the physical classroom context (e.g., “in this class”, “my classmates”) were replaced with phrases indicating an online or digital context (e.g., “my peers online”, “in our digital interactions”). Subjective experiences during the past month were evaluated (19). Participants indicated their agreement with each statement on a five-point Likert scale from (1 = “Never” to 5 = Always). A total score was calculated as the mean of all five items, with higher scores indicating greater perceived digital peer support. The adapted scale demonstrated excellent internal consistency in our sample, with a McDonald's Omega (**ω**) = 0.89.

#### 2.3.2 Depressive symptoms

Depressive symptoms over the prior two weeks were assessed with the 9-item Patient Health Questionnaire (PHQ-9) (16, 17). Items are scored from 0 (“Not at all”) to 3 (“Nearly every day”). The total score ranges from 0 to 27, and its categories are as follows: 0–4 (minimal), 5–9 (mild), 10–14 (moderate), 15–19 (moderately severe), and 20–27 (severe). The PHQ-9 has been widely validated and exhibits excellent psychometric properties in both clinical and non-clinical samples, with validation also for our specific linguistic context (20). Scale reliability: in this study, the sample of the scale was found to have high internal consistency (Cronbach's α = 0.88).

#### 2.3.3 Anxiety symptoms

The 7-item Generalized Anxiety Disorder scale (GAD-7) was employed to assess anxiety symptom severity over the preceding two weeks (17). Items are scored from 0 to 3, yielding a total score of 0–21, categorized as minimal (0–4), mild (5–9), moderate (10–14), and severe (15–21). The GAD-7 is a widely recognized and validated (21), and demonstrated excellent reliability in our study (α = 0.90).

#### 2.3.4 Psychological distress

The 6-item Kessler Psychological Distress Scale (K6) was used to measure general distress over the past month (22). Items are rated on a five-point scale from 1 (“None of the time”) to 5 (“All of the time”). The total score (range 6–30) was used as a continuous variable, with established cut-off values indicating moderate (9–12) and serious (13–24) distress. The K6 is a robust screening instrument globally validated by (23), and it had good reliability in our data (α = 0.85).

#### 2.3.5 Physical health

The single-item self-rated health question was used to measure overall physical health (“In general, would you say your physical health is excellent/stable/poor?”). Responses were scored on a five-point Likert scale (1 = “very poor”; 5 = “excellent”). This single-item measure is a well-established and strong predictor of objective health outcomes, functional decline, and mortality in epidemiological research, justifying its use for broad correlational analysis in this context.

### 2.4 Data analysis plan

Descriptive statistics and all statistical analyses were performed using IBM SPSS Statistics (version 28) software. These analyses were conducted in multiple stages: descriptive statistics in the form of frequencies, percentages, means, and standard deviations with regard to the attributes of the study sample and distribution of all major variables. Bivariate analyses, including Preliminary Pearson's and Spearman's (depending on data normality), were conducted to examine the unadjusted relationships between the digital peer support score, mental health outcomes, and covariates. Multivariate inferential analyses, to address the study's primary aim and control for potential confounders, a series of multivariate regression models was constructed. Linear regression models are used for continuous outcome variables (PHQ-9 total score, GAD-7 total score, K6 total score). The digital peer support score was entered as the primary predictor, with age, gender, socioeconomic status, and health history included as covariates.

Logistic regression models, used for binary clinical outcomes derived from the scales, such as the presence of moderate-to-severe depression, (PHQ-9 ≥10 vs. < 10). Similarly, models were adjusted for the same set of covariates. For all regression models, we report unstandardized (B) and standardized (β) coefficients for linear regression, and Odds Ratios (OR) with 95% Confidence Intervals (CI) for logistic regression. The significance level was *p* < 0.05, two-tailed. Model fit indices (R^2^, adjusted *R*^2^) are presented, if applicable. This study fills an important gap in understanding from the perspective of students how digital peer support interventions impact physical and mental health and overall wellbeing, a population subgroup about which little is known from previous research. The quality and consistency of the results will be enhanced by this methodological rigor, which will also make it possible to conduct scientifically sound research on peer support treatments for mental and physical health.

## 3 Results

This section begins with descriptive statistics of the study variables, followed by conditions to be satisfied for the research model testing to proceed, and presents the sample relationships among all measures (correlation matrix). The socio-demographic information offers a picture of the characteristics of respondents, including age, gender, year of study, personal monthly household income, and residency status. The socio-demographic profile of the 230 undergraduate students included in this cross-sectional study is shown in Table 1. The median age in the past year's cohort was 24 years (IQR: 21–27), and most participants (41.3%) were aged 22–25. There were slightly more men (54.3%) than women in the sample (45.7%). More than half of the students (51.3%) reported a monthly personal income in the lowest category range (0–25,000 PKR, approximately $0–$90 USD), which indicates that at least half of the sample's financial characteristics support the generalizability and reliability of the digital peer support intervention tested.

**Table 1:** Socio-demographic characteristics of the participants.

Variable	*N* (%)
Total	230
Age	
18–21	58 (25.2)
22–25	95 (41.3)
26–29	55 (23.9)
30–33	22 (9.6)
Median (IQR)	24 (21–27)
Gender	
Male	125 (54.3)
Female	105 (45.7)
Year of study	
Preparatory year	13 (5.7)
First year	48 (20.9)
Second year	39 (17.0)
Third year	43 (18.7)
Fourth year	34 (14.8)
Fifth year	30 (13.0)
Internship	23 (10.0)
Personal monthly income (PKR)	
0–25,000 PKR ($0–$90 USD)	118 (51.3)
25,001–50,000 PKR ($91–$180 USD)	73 (31.7)
More than 50,000 PKR (> $180 USD)	39 (17.0)
Residence status	
With family	180 (78.3)
Home alone	50 (21.7)
Digital peer support	
Higher support	148 (64.3)
Lower support	82 (35.7)

Table 2 presents the distribution of participants across levels of Self-rated Health (SRH) and anxiety symptoms, with anxiety assessed using the Generalized Anxiety Disorder 7 (GAD-7). Physical health was measured with Self-rated Health (SRH) as the dependent variable, and they were instructed to rate their overall physical health (17). The responses showed that 22 individuals (9.6%) reported destitute physical health, while the largest group, consisting of 108 individuals (47%), reported a fair state of physical health. A smaller percentage, 72 (31.3%), reported good physical health, and literally 28 (12.2%) described physical health as excellent. Such findings describe participants' subjective assessment of their general physical health, using a self-reported rating scale, with no symptom-based measure of physical health being included.

**Table 2:** Classification of self-rated general physical health (single-item SRH) and anxiety/depression severity (GAD-7) among undergraduate university students (*N* = 230).

Category	*N*	Percentage (%)
Self-rated general physical health		
Poor	22	9.6
Fair	108	47
Good	72	31.3
Excellent	28	12.2
Anxiety and depression		
Minimal (0–4)	17	7.4
Mild (5–9)	90	39.1
Moderate (10–14)	71	30.9
Severe (15–21)	52	22.6

The severity of anxiety symptoms was determined using the GAD-7 scale, a tool assessing mental health. The GAD-7 scale classifies individuals into four levels of anxiety intensity. Seventeen participants (7.4%) exhibited minimal anxiety symptoms (GAD-7 score 0–4), while 90 participants (39.1%) exhibited mild anxiety symptoms (GAD-7 score 5–9). A significant number of participants, 71 (30.9%), exhibited moderate anxiety symptoms (GAD-7 score 10–14), while 52 (22.6%) presented severe anxiety symptoms (GAD-7 score ≥15). These results imply that the prevalence of anxiety symptoms might be very high, as 92.6% of participants showed some level of anxiety. Half of the sample (53.5%) reported moderate to severe levels of anxiety symptoms.

Table 3 presents the univariate analysis of factors associated with lower self-rated physical health. The results indicate that among the socio-demographic variables assessed, only personal monthly income and digital peer support were significantly associated with self-rated physical health categories. A significant association was found for personal income (χ^2^ = 6.112, *p* = 0.045). Students in the middle-income bracket (25,001–50,000 PKR, approximately $91–$180 USD) constituted 44.4% of the group reporting lower self-rated health (Poor/Fair), which is higher than their proportion in the higher health-rated group (26.3%). This suggests a notable association between mid-level income and poorer self-rated health in this sample.

**Table 3:** Univariate analysis of factors associated with self-rated general physical health among undergraduate university students (*N* = 230).

Variable	*N* (%)	Lower self-rated health (poor/fair) *N* (%)	Higher self-rated health (good/excellent) *N* (%)	*X* ^2^	*p*-value
Total	230	135 (58.7)	95 (41.3)		
Age				5.024	0.192
18–21	50 (21.7)	27 (20.0)	23 (24.2)		
22–25	88 (38.3)	58 (43.0)	30 (31.6)		
26–29	60 (26.1)	33 (24.4)	27 (28.4)		
30–33	32 (13.9)	17 (12.6)	15 (15.8)		
Gender				2.543	0.111
Female	110 (47.8)	60 (44.4)	50 (52.6)		
Male	120 (52.2)	75 (55.6)	45 (47.4)		
Year of study				7.142	0.331
Preparatory year	17 (7.4)	6 (4.4)	11 (11.6)		
First year	50 (21.7)	28 (20.7)	22 (23.2)		
Second year	35 (15.2)	19 (14.1)	16 (16.8)		
Third year	38 (16.5)	24 (17.8)	14 (14.7)		
Fourth year	40 (17.4)	26 (19.3)	14 (14.7)		
Fifth year	30 (13.0)	15 (11.1)	15 (15.8)		
Internship	20 (8.7)	17 (12.6)	3 (3.2)		
Personal monthly income (PKR)				6.112	0.045
0–25,000 PKR ($0–$90 USD)	102 (44.3)	55 (40.7)	47 (49.5)		
25,001–50,000 PKR ($91–$180 USD)	85 (37.0)	60 (44.4)	25 (26.3)		
More than 50,000 PKR (> $180 USD)	43 (18.7)	20 (14.8)	23 (24.2)		
Residence status				0.217	0.642
With family	172 (74.8)	105 (77.8)	67 (70.5)		
Home alone	58 (25.2)	30 (22.2)	28 (29.5)		
Digital peer support				4.761	0.029
Higher support	160 (69.6)	100 (74.1)	60 (63.2)		
Lower support	70 (30.4)	35 (25.9)	35 (36.8)		

Digital peer support also showed a significant association (χ^2^ = 4.761, *p* = 0.029). Students reporting higher levels of digital peer support were more likely to rate their physical health positively. Specifically, 74.1% of students with high support were in the lower self-rated health category, indicating they represented a larger proportion of those reported health concerns, while 63.2% of students with lower support were in the higher health-rated group. This points to a complex relationship where higher digital support is more prevalent among those acknowledging health issues. In contrast, age, gender, year of study, and residence status showed no statistically significant associations with self-rated physical health all (*p* > 0.05).

The results of the univariate analyses examining factors associated with anxiety and depression are presented in Table 4. Among the socio-demographic variables assessed, personal monthly income and digital peer support were the only factors found to have a statistically significant association with mental health outcomes. Students with the lowest personal income (PKR-0–25,000 PKR, approximately $0–$90 USD) constituted 47.8% of the sample but comprised a disproportionately higher percentage (57.8%) of the group experiencing elevated anxiety and depression, compared to 41.4% of the group with lower symptom (χ^2^ = 6.901, *p* = 0.038). This indicated that lower economic status was significantly associated with poorer mental health. Furthermore, access to digital peer support showed a significant protective association. A majority of students (68.7%) reported higher levels of digital peer support. Within this group, 74.3% were in the lower anxiety and depression category. In contract, among students with lower digital peer support, only 60.0% were the lower symptoms category, while 40.0% experienced higher symptoms (χ^2^ = 4.783, *p* = 0.026). Other variables, including age, gender, year of study, and residence status, were not significantly associated with anxiety and depression levels in this analysis (*p* < 0.05).

**Table 4:** Univariate analysis of factors associated with anxiety and depression among undergraduate university students (*N* = 230).

Variable	N (%)	Lower anxiety/depression *N* (%)	Higher anxiety/depression *N* (%)	*X* ^2^	*p*-value
Total	230	140 (60.9)	90 (39.1)		
Age				5.215	0.178
18–21	55 (23.9)	32 (22.9)	23 (25.6)		
22–25	98 (42.6)	64 (45.7)	34 (37.8)		
26–29	50 (21.7)	28 (20.0)	22 (24.4)		
30–33	27 (11.7)	16 (11.4)	11 (12.2)		
Gender				2.312	0.102
Female	120 (52.2)	65 (46.4)	55 (61.1)		
Male	110 (47.8)	75 (53.6)	35 (38.9)		
Year of study				6.945	0.329
Preparatory year	15 (6.5)	6 (4.3)	9 (10.0)		
First year	43 (18.7)	26 (18.6)	17 (18.9)		
Second year	38 (16.5)	23 (16.4)	15 (16.7)		
Third year	40 (17.4)	28 (20.0)	12 (13.3)		
Fourth year	37 (16.1)	25 (17.9)	12 (13.3)		
Fifth year	34 (14.8)	18 (12.9)	16 (17.8)		
Internship	23 (10.0)	14 (10.0)	9 (10.0)		
Personal monthly income (PKR)				6.905	0.038
0–25,000 PKR ($0–$90 USD)	110 (47.8)	58 (41.4)	52 (57.8)		
25,001–50,000 PKR ($91–$180 USD)	80 (34.8)	55 (39.3)	25 (27.8)		
More than 50,000 PKR (> $180 USD)	40 (17.4)	27 (19.3)	13 (14.4)		
Residence status				0.205	0.661
With family	176 (76.5)	108 (77.1)	68 (75.6)		
Home alone	54 (23.5)	32 (22.9)	22 (24.4)		
Digital peer support				4.783	0.026
Higher support	158 (68.7)	104 (74.3)	54 (60.0)		
Lower support	72 (31.3)	36 (25.7)	36 (40.0)		

Bivariate analyses (chi-square tests) were conducted to explore the association between socio-demographic factors, digital peer support, and mental health outcomes The practical significance of these association was assessed using Cramer's (Table 5). For self-rated general physical health, only digital peer support (χ^2^ = 4.761, Cramer's V = 0.144) and personal income (χ^2^ = 6.112, Cramer's V = 0.115) demonstrated statistically significant associations, with effect sizes in the small range. All other socio-demographic variables (age, gender, year of study, residence status) showed non-significant results with negligible or small effect sizes (*V* ≤ 0.105).

**Table 5:** Standardized effect sizes (Cramer's V) for associations between perceived digital peer support, socio-demographic factors, and health outcomes.

Association	χ^2^	*n*	*k*	Cramer's V	Interpretation
Self-rated general physical health					
Age	5.024	230	4	0.085	Negligible
Gender	2.543	230	2	0.105	Small
Year of study	7.142	230	7	0.072	Negligible
Income	6.112	230	3	0.115	Small
Residence status	0.217	230	2	0.031	Negligible
Digital peer support	4.761	230	2	0.144	Small (upper range)
Anxiety and depression					
Age	5.215	230	4	0.086	Negligible
Gender	2.312	230	2	0.106	Small
Year of study	6.945	230	7	0.071	Negligible
Income	6.905	230	3	0.122	Small
Residence status	0.205	230	2	0.03	Negligible
Digital peer support	4.783	230	2	0.144	Small (upper range)

For anxiety and depression, a similar pattern emerged. Digital peer support (χ^2^ = 4.783, Cramer's V = 0.144) and personal income (χ^2^ = 6.905, Cramer's V = 0.122) were again the only factors with statistically significant associations and small effect sizes. The associations for all other variables were non-significant with negligible effect sizes (*V* ≤ 0.106). To explore the independent contribution of each variable while adjusting for others, multivariable binary logistic regression was conducted. The models accounted for a modest portion of the variance in outcomes: 7.8% (Nagelkerke *R*^2^ = 0.078) for anxiety symptoms and 8.9% (Nagelkerke *R*^2^ = 0.089) for depressive symptoms. After controlling for age and gender, both digital peer support and low personal income remained significant independent predictors.

Students with lowest personal income had over twice the odds pf moderate-to-severe anxiety (AOR = 2.10, 95% CI = 1.05–4.21, *p* = 0.036) and depression (AOR = 2.25, 95% CI = 1.12, 4.25, *p* = 0.023). Conversely, higher digital peer support was associated with significantly lower odds, corresponding to a 45% reduction in the odd's substantial anxiety (AOR = 0.55, 95% CI = 0.31, 0.97, *p* = 0.039) and a 48% reduction for depression (AOR = 0.52, 95% CI = 0.29, 0.93, *p* = 0.028). While bivariate effect sizes were small, multivariate analysis conforms that lower socioeconomic status is a significant and independent risk factors and digital peer support is a significant independent protective factor for student mental health, even after according for other demographic variables.

## 4 Discussion

In this cross-sectional study, we found significant associations between perceived digital peer support and mental health outcomes among university students. After adjusting for income and demographic characteristics, higher perceived digital peer support was associated with lower odds of reporting moderate-to-severe anxiety (AOR = 0.55) and depression (AOR = 0.52). These results contribute to a growing literature acknowledging the relevance of digitally facilitated peer interaction for student wellbeing (13). But it must be emphasized that our method discovers connections and not causal effects. Unobservable confounders, such as the general tendency of students to engage in self-care, may lead to the perception that both seeking support and experiencing better mental health are related, thereby clarifying the conditions under which these associations occur. The study is also a reminder about the importance of socioeconomic position; low personal income remained as a robust predictor of elevated anxiety and depression odds, suggesting that digital resources are closely linked to wider health structural determinants.

However, we observed a slightly negative effect in our study (AORs 0.5–0.6) compared to formal peer support reviews is astonishing. For instance, Yeo et al. (14) highlighted the role of structure and trained facilitators to deliver digital support for chronic physical health conditions. Our results suggest that informal (perceived) peer support in typical digital contexts may still be related to mental health among a non-referred, student population (10). The difference could be explained by the sample population; it is conceivable that students view more informal or online peer support as more readily available and less stigmatizing than a traditional program, and therefore interest is easier.

Furthermore, the slightly effect sizes are consistent with meta-analyses like Lloyd-Evans et al. who described the factors involved and sometimes moderate effects of peer support in physical and mental health (24). Previous studies such as ours have found that the effect sizes were statistically significant and turned out to be nearly trivial, indicating that perceived digital peer support is part of a constellation of factors related to wellbeing. The more likely positive associations may work via avenues reported in the literature where digital platforms provide fora, inter alia, for self-disclosure with less stigma and possibly reduced emotional distress (25), as well as a compact space to deliver practical, peer-learned coping strategies that can enhance one's own sense of efficacy (26).

The present study indicates that such supporting mechanisms, co-mechanisms, or variations of them (27) may take place within the shared digital ecosystem of apps and social media with affordances such as synchronicity (27). Our results also did not confirm the expectation that demographic variables such as age or gender moderated the link between peer support and mental health outcomes. The findings may suggest the association as a general phenomenon among subgroups of students, but further replication in larger and more diverse samples is required.

## 5 Implications of the study

These findings suggest that a particular developmental association exists, which may be of relevance to wellbeing strategies in universities. University administrations and student support services might want to consider evaluating and promoting environments in which it is perceived that positive digital peer support is available. Instead of displacing professional service, acknowledging the relationship to better mental health outcomes upholds a scalable, low-threshold resource that is part of students' social network. The strong relationship with income inequality also suggests that universities could combine such promotional activity with wider drives to address socioeconomic inequalities, including but not limited to improving financial support and access to the necessary resources. This study highlights the case for further research. Longitudinal studies are needed to explore the temporal relationships between perceived support and mental health. To go beyond association to causal inference, an experimental or quasi-experimental design is required. Use of multi-item, validated measures for all major constructs would have increased depth and validity. Finally, cross-cultural and multi-institutional research that would allow testing the generalizability of these associations and considering potential cultural moderators of this association.

## 6 Limitations

There are several methodological limitations that the present study has brought to light. As the current investigation is a cross-sectional study, we could not infer causation and draw conclusions about unmeasured confounding. The data on mental health and perceived support are self-reported, with the risk of recall and social desirability bias. The convenience sampling from one university also restricts the generalizability of the findings to other students' samples or cultures. A single self-rated measure of physical health is a coarse measure that lacks the precision of a multi-item validated scale or an objective index of physical health. Most importantly, a significant limitation is inherent in the study design: we were not carrying out an intervention study. The exposure variable was perceived digital peer support rather than delivered intervention. We cannot, hence, describe what specific digital platform was used, the frequency or type of interactions, the existence of trained facilitators, or anything regarding “intervention fidelity”. This seriously limits our ability to understand how or why perceived support is linked with mental health and precludes recommendations for program design.

## 7 Conclusion

The results of this study indicate a high prevalence of at least moderate anxiety among undergraduate students and the success of health interventions in improving the physical and mental capacities. This article adds to an increasing body of literature focused on digital peer support, which has generally been restricted to clinical (rather than non-clinical) samples. These conclusions represent purely mechanistic interpretations of how the theoretical “modes of action” we assume will have been enacted, and an interesting limitation in our results suggests a research direction worth pursuing. The research was conducted with just a few groups, so more work is needed to understand how digital peer support might function for a larger swath of people with complex health problems. Although some positive outcomes were reported for physical and mental health, additional research is required on the sustainability of interventions and processes by which peer support might impact people's lives, particularly in an online context.

This study adds to the emerging evidence base that digital peer support might be beneficial for health and wellbeing in a range of different contexts. It also comes with many interesting questions left unanswered. For instance, the studies included also explore whether platform characteristics like interactivity, perceived ease of use, and anonymity might have a role in the effects of peer support interventions. The study looked at demographic antecedents (age, socioeconomics, and culture) of such an audience for a platform with digital stories and tested per treatment whether giving access to online peer support affected engagement as anticipated. Completing these missing components would maximize the opportunity to implement what works in effective practices for wider dissemination and reach. For example, we considered how well the results of this study correlate with previous literature and how they will help shape our understanding of the use and experience of digital peer support among different population groups. The results are important for health policy, as they offer new pathways to consider the use of peer support in people's health and social care. The research lays the groundwork for future efforts to enhance the effectiveness of digital interventions aimed at promoting public health across diverse cultural backgrounds and social groups.

## Data Availability

The raw data supporting the conclusions of this article will be made available by the authors, without undue reservation.
